# Qatar's National Mental Health Study—The World Mental Health Qatar

**DOI:** 10.1002/mpr.2008

**Published:** 2024-05-10

**Authors:** Salma Mawfek Khaled, Majid Al‐Abdulla, Iain Tulley, Sheik Mohammed Al‐Thani, Peter W. Woodruff

**Affiliations:** ^1^ Department of Population Medicine College of Medicine Qatar University Doha Qatar; ^2^ Department of Psychiatry Hamad Medical Corporation Doha Qatar; ^3^ College of Medicine Qatar University Doha Qatar; ^4^ Department of Public Health Ministry of Public Health Doha Qatar; ^5^ School of Medicine and Population Health University of Sheffield Sheffield UK

**Keywords:** Composite International Diagnostic Interview (CIDI), epidemiology, prevalence, psychiatry, Qatar

## Abstract

**Background:**

We provide an overview of Qatar's first epidemiological study on prevalence, predictors, and treatment contact for mood and anxiety disorders.

**Aims:**

We highlight the importance of the three‐pronged study, its aims, and its key components.

**Materials & Methods:**

The first component comprised a probability‐based representative survey of Qatari and non‐Qatari (Arab) adult males and females recruited from the general population and interviewed using the International Diagnostic Interview (CIDI version 3.3). The second component, a clinical reappraisal study, assessed concordance between diagnoses based on the CIDI and independent clinical assessments conducted by trained clinical interviewers. The third component comprised a resting‐state functional magnetic resonance imaging study of healthy survey respondents who were matched to patients with psychosis.

**Results:**

5000 survey interviews provided data on prevalence and treatment of common mental disorders. Clinical re‐interviews (*N* = 485) provided important diagnostic validity data. Finally, state‐of‐the art structural and functional brain markers for psychosis were also collected (*N* = 100).

**Discussion:**

Descriptive epidemiological data were collected to inform future mental health priorities in Qatar and situates these within a global context.

**Conclusion:**

The study fills important gaps in regional and global estimates and establish necessary baseline to develop comprehensive risk estimates for mental health in Qatar’s young population.

## 1 THE WORLD MENTAL HEALTH QATAR (WMHQ) STUDY

To date, the only available data on psychiatric morbidity in Qatar comes from primary care settings with a paucity of nationally representative epidemiological data on prevalence, predictors, and treatment contact of mental disorders in the general population. Better information is necessary for the purposes of service planning, implementation, and evaluation of policy as well as community‐based interventions. The lack of a national capacity to conduct population mental health research is a major challenge. The World Mental Health (WMH) Survey Initiative has been instrumental in supporting infrastructure to collect comprehensive and representative psychiatric epidemiological data in different countries (Kessler et al., 2006). Therefore, we wished to establish a study in Qatar that would benefit from this initiative and to place our data within a global context.

The World Mental Health Qatar (WMHQ) is the first national general population study of psychiatric and physical morbidity in Qatar. Its aims are to establish the nation's capacity to conduct research in population mental health and support the initiation of a psychiatric epidemiological research program by establishing robust baseline estimates of the lifetime and 12 months prevalence of common psychiatric disorders. The study aimed to provide much‐needed baseline data for Qatar to be used for ongoing benchmarking and monitoring of its population's mental health. In addition, the WMHQ provides Qatar with an opportunity to collaborate in cross‐national comparative studies with other countries in the WMH consortium.

The WMHQ consisted of three main components. The first component was a national survey of probability‐based sample of Qatari and non‐Qatari (Arab) males and females age 18 years and above from Qatar's general population. A total of 5000 survey interviews were completed by our WMHQ certified team of researchers using computer‐assisted telephone interviewing (CATI) method of data collection (Khaled et al., 2024). The Composite International Diagnostic Interview (CIDI version 3.3) was previously adapted (Khaled et al., 2021) and administered during these interviews to assess the prevalence and burden of mental illness based on the fifth edition of the Diagnostic and Statistical Manual of Mental Disorders or DSM‐5 (American Psychiatric Association, 2013). Lifetime, 12‐month, and 30‐day prevalence of mood, anxiety, and stress‐related disorders were assessed in the WMHQ. In addition, information on age of onset, persistence, severity, treatment contact, daily function, physical comorbidity, and risk factors were also collected during the course of these interviews.

The second component comprised a clinical reappraisal, which was carried out using telephone follow‐up interviews of a subset of the original survey interviews (*n* = 485) to assess the diagnostic consistency of core modules of the newly translated and adapted Arabic version of the CIDI against independent clinical diagnoses by trained clinicians based on the Structured Clinical Interview for DSM‐5 (Amro et al., 2023). Results from the clinical validation component of this study constitute the basis of another paper in this issue (Khaled et al., 2023).

The third component of the study aimed to compare structural and functional brain markers for psychosis to healthy groups with varying trait‐based risk levels for the illness using resting‐state functional Magnetic Resonance Imaging (fMRI) technology. These healthy controls were a subset of the original sample who completed the survey component and were followed up and recruited for the fMRI component of the study. Healthy controls were matched on age and gender of patients with psychotic disorders who were recruited from the Psychiatry Department at Hamad Medical Corporation (HMC), the main national healthcare provider in Qatar. Both patients and controls were scanned using the same technology and procedure at Sidra Medicine. The findings from this component of the study will be published separately potentially providing major evidence forward in mapping biological risk preceding serious mental illness and those at early stages of risk thus informing services for early prevention efforts.

In this issue, the focus will be on describing the methodological aspects, clinical reappraisal, and main epidemiological findings of the survey component of the WMHQ study. The primary aim of the survey is to estimate the prevalence and associated factors of common mental disorders in Qatar's general population as this data were not previously available. These data are particularly important to combat stigma, which may minimize the perception of the importance of mental health generally as has been highlighted repeatedly in the literature (Fekih‐Romdhane et al., 2023; Kehyayan et al., 2021; Mahgoub et al., 2022; Zolezzi et al., 2017).

In this overview paper, we provide a brief background on Qatar as a country—including its history, recent sociopolitical events, and health challenges. We then provide an overview of developments of the mental health service in the country over the years in relation to the National Mental Health Strategy and in a global context. We review some of the main challenges in meeting the requirements of the strategy to make mental healthcare more accessible with reference to migrants' and women's mental health and the response to the COVID‐19 pandemic. Finally, we outline some community‐based research on mental illness in Qatar's population.

## 2 QATAR'S HISTORY, CULTURE, AND SOCIOECONOMIC TRANSFORMATION

The state of Qatar is a peninsula with a total area of 11,437 km^2^, located on the western coast of the Arabian Gulf. It consists of an estimated total population of 2.9 million people in 2022. Only 11.8% of these people are Qataris (Planning and Statistical Authority, 2021). The majority of the population are migrant residents from different countries: South Asia (India, Nepal, Bangladesh, Pakistan), South East Asia (the Philippines), and Arabs (Egypt, Sudan, Jordan, Palestine, Lebanon, and Iraq). Qatar consists of a total of nine municipalities, Doha is the capital and the most populous (Hukoomi, 2023). Despite its small area, Qatar is one of the richest countries in the world, as it is the second‐largest exporter of natural gas and has the world's third‐largest proven natural gas reserve (Hukoomi, 2023).

Sheikh Jassim bin Mohammed bin Thani was the founder of the state of Qatar and designated leader when the British forces exited in 1878 (Amiri Diwan, 2023). Since 1971, Qatar has been fully independent of the British Protectorate and has been ruled by the Al‐Thani family (Ministry of Foreign Affairs, 2022). Sheikh Tamim bin Hamad bin Khalifa Al‐Thani has been the Emir of the country since 2013 (Ministry of Foreign Affairs, 2022).

Islam is the dominant religion in Qatar, where 65% of the population identifies as Muslim (Central Intelligence Agency, 2023). As a result, customs, traditions, and the Islamic faith are all a part of Qatari culture, where Islam exerts a significant impact on peoples' behavior (Al‐Hamar et al., 2010).

Over the past 20 years, Qatar invested heavily in infrastructure development and growth. Qatar was the first Middle Eastern country to host the FIFA World Cup 2022. To prepare for this big event, Qatar built stadiums that are environmentally friendly and architecturally innovative (Hukoomi, 2022). Preparation for the World Cup required extensive construction and rapid expansion of airports, hotels and resorts, and improved roads and highways (El‐Sabek, 2017; Wiedmann et al., 2013).

In recent years, Qatar has also invested in healthcare by expanding and building new hospitals, primary care centers, and wellbeing centers (Al Khal et al., 2020). This construction program has relied heavily on migrant workers, predominantly from low‐income countries in the Asian sub‐continent. The vast majority are male laborers, with little or no vocational training, who are employed in the construction sector or oil‐and‐gas industry. Typically, they live with other males in accommodation provided by their employer. Prolonged separation from family and friends naturally places some of these migrant laborers under particular stress resulting in the need to access mental health services.

According to an earlier nationally representative study conducted in Qatar, migrants typically experience higher depressive symptoms than non‐migrants (Khaled & Gray, 2019). Among labor migrants, experiencing a problem with one's current employer in the last 3 months was significantly associated with depressive symptoms (Khaled & Gray, 2019). The study also showed that culture of origin was significantly associated with depressive symptoms with perceived quality of life, but not length of stay, an important variable in explaining differences in depressive symptoms between some cultural groups in Qatar (Khaled & Gray, 2019). The challenge for the Government funded healthcare system is to meet the needs of this large segment of the population, to identify the many and varied social determinants of mental health, and the clinical presentations that may arise from these disparate populations as well as try to prevent them.

As outlined in the Qatar National Vision 2030 document, the country aspires to transform itself into a knowledge‐based economy, moving away from its reliance on petrochemicals. This diversification of the economy and investment in human capital is intended to support growth and provide a high quality of living to all residents (Scharfenort, 2012). Increased socioeconomic status for Qataris corresponds with economic expansion that commenced in the 1970s (Al‐Hammadi & Alkaabi, 2021). Following its independence, Qatar launched a wide range of social welfare initiatives, such as university education, housing allowances, free health care, and reduced utility costs (Department of Social Development & General Secretariat for Development Planning, 2011; International Trade Administration, 2022; Ministry of Foreign Affairs, 2023; The Peninsula, 2022). A higher standard of living has also been achieved as a result of advancements in road networks, utility services, sewage treatment, and water desalination (Lawler et al., 2023). In addition, many institutions and social programs have been developed to provide assistance to low‐income households and people with disabilities (Department of Social Development & General Secretariat for Development Planning, 2011).

In June of 2017, the United Arab Emirates, Saudi Arabia, Egypt, and Bahrain cut diplomatic relations with Qatar marking the start of three‐and‐half year economic and political blockade. Qatar suffered tremendous financial losses during the blockade, yet it was able to handle the crisis effectively and was a coming of age moment for the country (Antoniades et al., 2021). The blockade was an important driver for transformation, forcing Qatar to exert its independence and preparedness for future challenges including: food and water self‐sufficiency and border security (Albasoos et al., 2021). It also improved cooperation and quality of policymaking across important sectors of the nation (Antoniades et al., 2021). The blockade also had the effect of strengthening nationalism, solidifying social identity and social cohesion of the Qatari population and its residents.

The investment in healthcare infrastructure and crisis readiness of the country post‐blockade helped prepare Qatar for the COVID‐19 pandemic. The COVID‐19 fatality rate in Qatar was among the lowest worldwide. This was partly due to the government's prompt and thorough efforts, which included modifying public health policies affording the implementation of a continuous mobile app surveillance system, COVID‐19 awareness, strategic testing, and providing free vaccinations to the entire population (Al Khal et al., 2020).

## 3 OVERVIEW OF QATAR'S MENTAL HEALTH SYSTEM

Over the past 3 decades, Qatar has invested heavily in developing its healthcare system and achieved parity with developed countries on many health indicators (World Health Organization, 2018). Qatar has invested heavily in improving and expanding its domestic healthcare provision. The mental health service has benefited from this expansion. The healthcare system in Qatar is government funded and regulated by the Ministry of Public Health. Health services are provided by a mix of public, private and semi‐government providers, with public providers constituting most of the healthcare activity across the country (Ministry of Public Health, 2018). Hamad Medical Corporation (HMC) is the main non‐profit healthcare provider in the country offering free services at the point of delivery to all residents of Qatar. In addition to establishing a national ambulance service and a home healthcare service, HMC now boasts six highly specialized hospitals including the Women's Hospital and Psychiatric Hospital (Ministry of Public Health, 2018).

## 4 MENTAL HEALTH STRATEGY AND INVESTMENT

**FIGURE 1:**
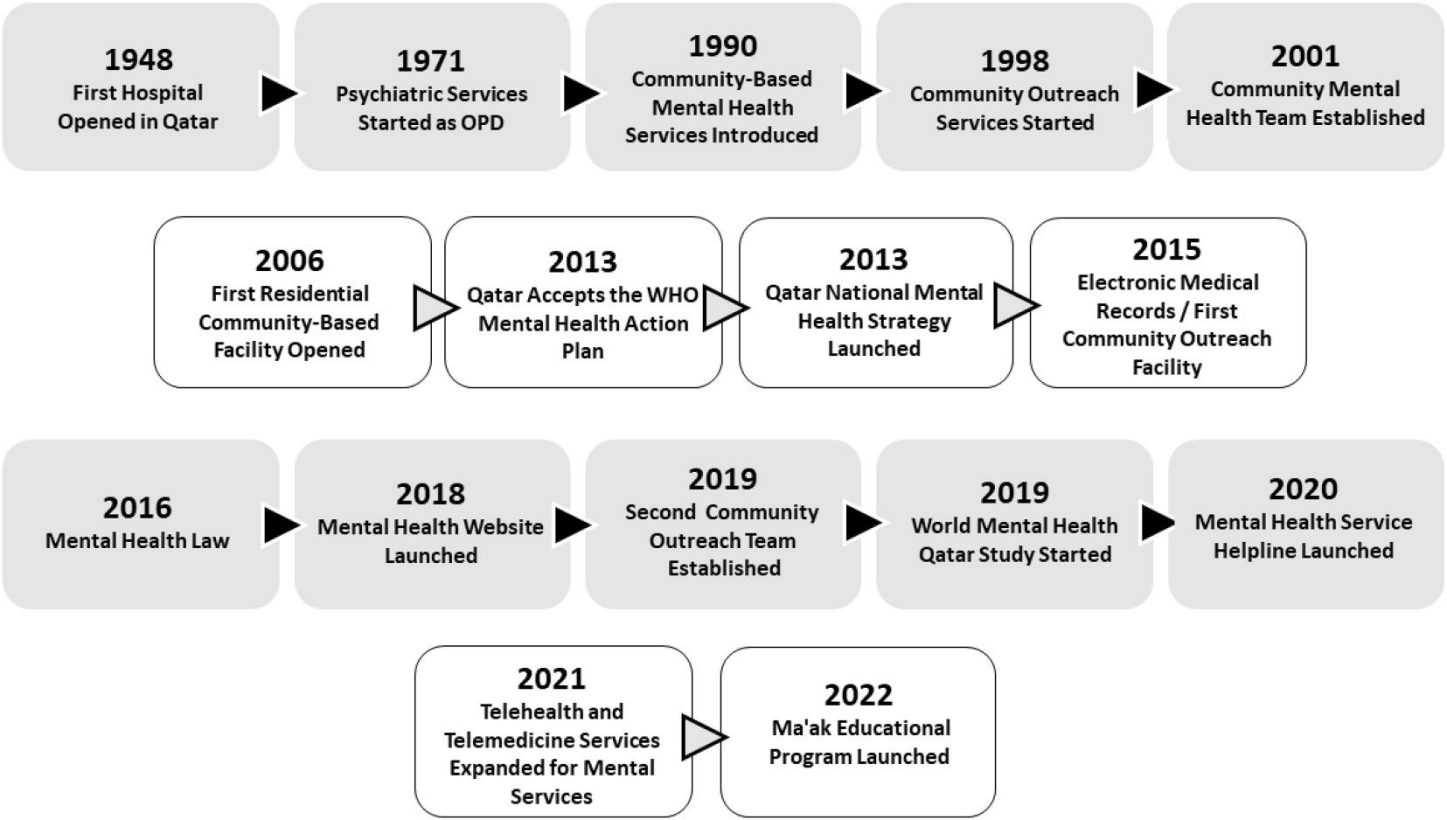
Summary of Qatar mental health service milestones.

Figure 1 summarizes the key milestones in the development of the Qatar's Mental Health Service to the present day after the first hospital was built in 1948 and the first psychiatric outpatient service started in 1971. Following the global trend to deliver mental healthcare in the communities where patients live, Qatar opened up its first community‐based service, outreach service and community‐based teams over the next several decades. This provided the impetus to set up the first residential community facility, and later, services more able to respond to community need such as the community outreach teams.

The Qatar National Mental Health Strategy, *“Changing Minds*, *Changing Lives”* (2013–2018) was pivotal in highlighting the importance of mental health. It set the direction for further investment in developing the mental health service under the umbrella of Public Health. This strategy provided the framework within which the mental health system could progress and, in doing so, focused on the following four overarching objectives: mental health promotion and prevention, provision of comprehensive, integrated services, strengthening mental health leadership and governance, and the improvement of information systems, research and evidence‐based practice (Ministry of Public Health, 2019).

The recognition that research was crucial to inform promotion and prevention as well as provide the basis for evidence‐based practice was further underscored by the Qatar National Mental Health & Wellbeing Strategic Framework (2019–2022). Additionally, mental health was given a boost when it was designated a key priority within the Public Health Strategy (2017–2022). Improved data on the nation's mental health was the catalyst for these strategic milestones.

## 5 DEVELOPMENTS IN MENTAL HEALTH PROVISION AND THE WMHQ

Over the course of the WMHQ and in parallel, there were certain initiatives that improved access to mental health provision. For instance, access to data improved with the introduction of electronic medical records, a Mental Health Website and the Mental Health Service Helpline, Telehealth and Telemedicine Services. These initiatives helped make the mental health service more accessible thus contributed to bringing help closer to people in the community who needed it and who might have previously been reluctant to seek help.

Subspeciality services were developed to better assist the patients, for example, forensic psychiatry, women mental health, child and adolescent psychiatry, old age and most recently services for patients with learning disabilities. The adoption of this approach placed both the individual and their family as the focal objective of developing and delivering care and sought to minimize stigma and improve access to specialist evidence based care. These patient and family‐centered services sought to meet the specific needs of different demographics and helped to reduce stigma. Highly specialized western trained experts were appointed and fellowships were provided to develop local professionals in the aforementioned subspecialties (Alabdulla et al., 2023).

There are many reasons why the profile of mental disorders may differ in Qatar from other countries that might otherwise be considered valid comparators, such as Saudi Arabia and other Gulf States. As such, reliance on clinical populations can be deceptive because of selection biases that do not take account of morbidity ‘hidden’ in specific communities, especially those where stigma may make people more reluctant to seek help for mental illnesses.

Hence, the WMHQ study was set up in January of 2019, supported by HMC, to provide this data, as described in these series of papers.

## 6 MENTAL HEALTH PROVISION IN QATAR COMPARED TO GLOBAL CONTEXT

The WHO's Mental Health Atlas provided service provision information shown in Table 1 illustrating how Qatar compares with WHO regions in mental health service provisions per 100,000 against available mental health facilities (hospitals, psychiatric unit in general hospitals, and community residential units) in 2020. It can be seen that, in Qatar, provision of mental health hospital and community facilities, whilst being above the lowest, are still well below the average for high income group countries. Considering the available facilities, however, hospital and community admissions are relatively high. It is noteworthy that while the community beds represent about a fifth of high‐income countries, the admissions to community beds are about a half. This indicates that community mental health activity is relatively high in Qatar, which is consistent with the aims of the Mental Health Strategy.

**TABLE 1:** A Comparison of Mental Health Facilities, Beds, and Admissions per 100,000 population for Mental Health Hospitals, Psychiatric Unit, and Community Residential Unit in Qatar relative to World Health Organization (WHO) Regions and World Bank Income Groups in 2020.

	Qatar	WHO region			World bank income group	
		EMR	EUR	Global	Low	High
Mental health hospital						
Facilities	0.04	0.02	0.14	0.05	0.02	0.08
Beds	2.30	4.40	35.00	10.80	1.90	28.60
Admissions	39.37	32.20	275.00	71.80	14.30	150.70
Psychiatric unit in general hospital						
Facilities	‐	0.05	0.32	0.17	0.03	0.37
Beds	‐	1.20	12.30	2.50	0.40	15.20
Admissions	‐	15.30	220.60	43.00	6.70	271.30
Community residential						
Facilities	0.11	0.70	2.80	0.20	0.02	1.90
Beds	5.01	1.50	53.30	5.10	1.00	25.40
Admissions	13.42	1.40	22.30	4.00	1.20	26.40

Abbreviations: EMR, Eastern Mediterranean Region; EUR, European Region; WHO, World Health Organization.

*Source*: World Health Organization (WHO) Mental Health ATLAS, 2020.

One of the many advantages of the WMHQ survey is that it uses the same methodology as other countries across the world so prevalence figures can be compared. Hence, data shown in Table 1 can be placed in the context of prevalence data from the WMH surveys to inform relative population need for this developing country compared to other countries in the Eastern Mediterranean Region region, other WHO regions, and World Bank income groups.

## 7 MENTAL HEALTH WORKFORCE IN QATAR—COMPARATIVE DATA WITH REGIONAL AND WORLD BANK INCOME GROUPS

Workforce is of course vital for any healthcare provision. As shown in Table 2, the WHO's Mental Health Atlas provides information about Qatar's mental workforce and how it compares with the rest of the world. It is noteworthy that the total mental health professionals per 100,000 population has more or less doubled since 2014 from 13.45 in 2014, 14.78 in 2017 and 25.28 in 2020 (not shown in Table 2). This table shows that Qatar has developed its mental health workforce considerably. Of particular note is the expansion of mental health nurses (18 per 100,000 population compared with 29 in high income countries). Psychiatrists comprise about 25% of those in high‐income countries, which reflects recent investment in expansion of these numbers and strengthening their career structure (such as the introduction of a training fellowship program more likely to enable retention of trained psychiatry residents). Psychologists are at the lower end of the scale, but this profession is expanding as a clinical career structure is developed. Occupational therapists and pharmacists are relatively well represented. More social workers are needed particularly as the new Mental Health Law is implemented, and there are moves to strengthen their career structure mental health.

**TABLE 2:** Comparative data for mental health workforce in Qatar relative to countries in different World Health Organization (WHO) region and World Bank income group in 2020.

	Qatar		WHO region			World bank income group	
	Number of mental health professionals	Mental health professionals per 100,000 population	EMR^a^	EUR^a^	Global	Low	High
Mental health workforce							
Psychiatrist^b^	63	2.2	1.0	9.7	1.7	0.1	8.6
Mental health nurses	515	18.2	3.0	25.2	3.8	0.4	29
Psychologist	46	1.6	1.0	5.4	1.4	0.1	10.7
Social workers	14	0.5	0.4	2.0	0.7	0.1	2.9
Other specialized mental health workers^c^	78	2.8	0.7	3.2	0.5	0.1	4.1
Total mental health professionals	716	25.3	6.1	45.5	8.1	0.8	55.3

Abbreviation: WHO, World Health Organization.

^a^ EMR, Eastern Mediterranean Region; EUR, European Region.

^b^ Not including child & adolescent psychiatristn.

^c^ Other specialized mental health workers include occupational therapists, pharmacist, speech therapist.

*Source*: World Health Organization Mental Health ATLAS (2020).

## 8 OUTPATIENT SERVICES AND MENTAL HEALTH SERVICE VISITS IN QATAR OVERTIME

As shown in Table 3, HMC provided data that illustrate the actual types of outpatient activity and how that has changed from 2018 to 2022 as the Mental Health Service has expanded in Qatar. The pre‐booked patients and ‘walk‐in’ (New) and follow‐up (FU) patients are shown along with numbers who attended, or did not attend (DNA).

**TABLE 3:** Outpatient services Trends and Mental Health Service Visits in Qatar (2018–2022).

Year	Booked		Total	Attended		Total	DNA		Total	Walk‐in		Total	Total seen		Total	Seen				DNA new	DNA FU	DNA MHS
	New	FU		New	FU		New	FU		New	FU		New	FU		New to FU rate	New to FU ratio	Walk in %	Attended %	%	%	%
2018	6838	41,039	47,877	4399	32,255	36,654	2439	8784	11,223	399	7731	8130	4798	39,986	44,784	8.3	1:8	18	82	36	21	23
2019	8918	45,099	54,017	5636	36,304	41,940	3282	8795	12,077	201	6859	7060	5837	43,163	49,000	7.4	1:7	14	86	37	20	22
2020	9758	43,880	53,638	8038	38,595	46,633	1720	5285	7005	1123	19,122	20,245	9161	57,717	66,878	6.3	1:6	30	70	18	12	13
2021	10,953	48,523	59,476	9139	41,970	51,109	1814	6553	8367	1494	22,041	23,535	10,633	64,011	74,644	6.0	1:6	32	68	17	14	14
2022	11,222	51,120	62,342	8407	42,812	51,219	2815	8308	11,123	1625	23,750	25,375	10,032	66,562	76,594	6.6	1:7	33	67	25	16	18

Abbreviations: DNA, patients who did not attend; FU, follow‐up patients; NEW or “Walk‐in”, patients who attended without pre‐booked appointments..

*Source*: Mental Health Service, Hamad Medical Corporation.

It can be seen that there has been significant increase in demand between 2018 and 2022 (total seen 44,784 to 76,594), whereas the total DNAs have reduced (from 23% to 18%). Hence, the service is becoming more efficient and people are perhaps more willing to be seen. Of note also is that the number of follow‐ups seen has increased markedly (39,986 to 66,562) from 2018 to 2022.

## 9 QATAR'S MENTAL HEALTH SERVICE—FOCUS ON WOMEN'S MENTAL HEALTH

It is widely acknowledged in the literature that there are gender disparities in mental health and that mental illnesses have a higher burden on women compared to men (Kessler et al., 2005; Needham & Hill, 2010; Seedat et al., 2009). The National Health Strategy (2018–2022) in Qatar, emphasized the importance of the delivery of specialized services to women (Ministry of Public Health, 2021). Sidra Medicine is a tertiary care hospital in Qatar, which provides mental health services for women in the perinatal period and for children (Sidra Medicine, 2023). Perinatal mental health services are provided by high quality and skilled experts in this field whose aim is to prevent any deterioration of health during pregnancy and in the postnatal period (Sidra Medicine, 2020).

To address the risk of adverse mental health outcomes for women, Qatar has established gender‐specific interventions. In 2020, a committee for the development of women's mental health services was established, under the direction of a clinician and a nurse skilled in providing gender‐specific care for women in Qatar (Alabdulla et al., 2022). The development of gender‐specific mental health services for women was based upon recommendations documented in a report by this committee. Such developments include establishment of in‐patient services for women, training programs for staff from different sectors working with women, easy access through the setup of a Virtual Women's Mental Health Service and specialty clinics for women's mental health, collaboration with other organizations that provide services for women, and development of a governance plan that includes conducting epidemiological research (Alabdulla et al., 2022).

Currently, there is limited population‐based prevalence data available for common mental disorders and treatment contact among women in Qatar, which makes it hard to paint a clearer picture of the current mental health status for women in the country. The burden of mental illness may also vary in relation to women's reproductive phases. A recent study conducted in primary health care centers reported that the proportion of pregnant women with antenatal depression in Qatar was approximately 21% (Naja et al., 2021). Other studies also conducted in primary care reported the prevalence of post‐partum depression ranging from 18% (Burgut et al., 2013) to 23% (Deepu & Rupa, 2019).

In line with the above priorities, the WMHQ study provides baseline data on prevalence disparities on mood and anxiety disorders in Qatar and gender differences in treatment seeking across different mental health sectors.

## 10 QATAR'S MENTAL HEALTH SERVICE DURING COVID‐19 PANDEMIC

During the COVID‐19 pandemic, several restrictions were imposed, limiting in‐person visits to healthcare centers. These restrictions dramatically impacted the provision of mental health services and follow up compliance. Therefore, in order to reduce this adverse impact on mental health, services for telepsychiatry were introduced (Karim et al., 2020). Such services were provided via videoconferencing or telephone calls, to ensure prompt access to mental health services. Preliminary outcomes from the use of telepsychiatry are encouraging, and continued use of these services is essential given the rise in need for mental health care following the pandemic (Kehyayan et al., 2020). In addition to the telepsychiatry services, a helpline was established for urgent mental health cases (Gulf Times, 2020).

These advances in mental health provisions are important to take into account when interpreting 12‐month prevalence and other treatment‐seeking data. For instance, these advances in service provision impact on how we are able to compare estimates from the WMHQ collected during the COVID‐19 pandemic to data collected post COVID‐19 pandemic. Thus, the WMHQ offers unique population level data on the mental health status of the general population during this time and provides a snapshot of the performance of Qatar's mental health service during a global health crisis.

## 11 COMMUNITY‐BASED MENTAL HEALTH RESEARCH IN QATAR

The launch of the National Mental Health Strategy was signaled the state's recognition of mental health as a major public health concern. Raising awareness of mental health and minimizing stigma surrounding mental illnesses are included as key goals (Ministry of Public Health, 2019). To date, however, basic epidemiological data on the burden of mental illness in the general population is largely absent. For example, there is currently no baseline population‐based prevalence data for Qatari nationals and culturally similar Arab migrants.

According to a study conducted prior to the COVID‐19 pandemic, the point prevalence of any major depressive episode (including subthreshold episodes) ranged between 4.2% and 6.6% among the general population of Qatar (Khaled, 2019). Using the same methodology, the point prevalence of any generalized anxiety disorder was estimated by the same authors in another pre‐pandemic study to be 17.0% with an estimate of 3.6% for moderate and severe subtypes (Khaled & Zolezzi, 2021). Both studies reported an association between Arab ethnicity and higher rates of depression and generalized anxiety relative to other ethnicities (Khaled & Zolezzi, 2021).

Using data from these repeated nationally representative cross‐sectional surveys spanning 2017, 2018, 2020/2021, a pre‐ and post‐ COVID‐19 pandemic trend analysis was conducted by the same group to assess changes in prevalence of major depressive episodes and generalized anxiety disorder symptoms. The point prevalence of depressive symptoms was 6.6% in 2017 and 6.5% in 2020/2021. The point prevalence of generalized anxiety disorder symptoms was 3.6% in 2018 and 5.1% in 2020/2021. According to this study, the prevalence of depression and anxiety after the first wave of COVID‐19 did not differ significantly to pre‐pandemic estimates (Khaled et al., 2022). However, these studies had to rely upon the use of screening tools rather than structured interviews to assess depressive and anxiety symptoms (Khaled et al., 2022).

Except for a face‐to‐face pilot study (Khaled et al., 2021) that was conducted prior to the pandemic, the WMHQ study (including a second pilot) was adapted for phone methodology and conducted during the first and second waves of COVID‐19 pandemic. As mentioned in another manuscript published in this issue (Khaled et al., 2024), the CIDI instrument had to be adapted for phone mode by shortening the length of the interview. This was done using two main strategies including restricting the WMHQ diagnostic assessments of the CIDI to mood (major depression and bipolar I/II disorders) and anxiety disorders (generalized anxiety, posttraumatic stress, panic and obsessive‐compulsive disorders). Hence, we were able to make the study feasible for administration over the phone during the pandemic. We focused on this list of disorders in light of available literature showing that these two classes of mental illness accounted for most of the burden of disease in Qatar. For example, major depression and generalized anxiety disorders were the most prevalent psychiatric disorders among the primary healthcare population in Qatar (Bener et al., 2015; Ghuloum et al., 2011; Khaled, 2019). The reported lifetime prevalence estimates were 13.5% for depression and 10.3% for generalized anxiety disorder in Qatari nationals attending primary healthcare setting (Ghuloum et al., 2011). The lifetime prevalence for other disorders included obsessive compulsive (11.0%), posttraumatic stress (10.5%), panic disorders (10.3%) and bipolar disorder (4.3%) (Ghuloum et al., 2011). Notably, the prevalence of the aforementioned psychiatric disorders was significantly higher in women than in men (Ghuloum et al., 2011).

## 12 CONCLUSIONS

The WMHQ, as part of an international global collaboration, provides Qatar with internationally validated population‐based prevalence data to inform the direction and focus of community‐based mental health services. Studies have shown community platforms to be an effective alternative to hospital‐based care at expanding access to services. Based on the National Mental Health strategy recommendations, Qatar has implemented community outreach services to maximize medication adherence, prevent relapses, and reduce the need for in‐patient care. In order to prioritize services, however, population level prevalence data are needed to determine the extent and burden of mental illness in the general population. This study provides comprehensive descriptive data on the prevalence of common mental disorders. These findings that the WMHQ study provides help build up the evidence‐based data for planning services and to aid policy‐making by identifying the prevalence and risk factors, treatment gaps and outcomes in Qatar's indigenous Arab population. We focused on Arab residents (Qatari non‐migrants and non‐Qatari migrants) in the WMHQ because their sociodemographics are different from the other groups. Given similarities of culture and geographical location, findings from the WMHQ study is of direct relevance to the region and potentially fills gaps in global estimates that lack data from Qatar and most countries in the Middle East.

## AUTHOR CONTRIBUTIONS

**Salma Mawfek Khaled**: Conceptualization; investigation; funding acquisition; writing ‐ original draft; methodology; writing ‐ review & editing; data curation; project administration; supervision; validation; visualization; formal analysis. **Majid Al‐Abdulla**: Funding acquisition; writing ‐ review & editing; project administration; supervision; data curation. **Iain Tulley**: Writing ‐ review & editing; project administration. **Sheik Mohammed Al‐Thani**: Writing ‐ review & editing; project administration. **Peter W. Woodruff**: Conceptualization; funding acquisition; writing ‐ original draft; methodology; writing ‐ review & editing; supervision.

## CONFLICT OF INTEREST STATEMENT

None.

## ETHICS STATEMENT

Qatar University (QU‐IRB 1219‐EA/20) approved the study.

## Data Availability

The data that support the findings of this study are available from Dr. Salma M. Khaled, the principal investigator of the study at skhaled@qu.edu.qa, upon reasonable request and pending additional ethical approval.
